# Removal of an Embedded Taser Probe in the Glans Penis in the Operating Room

**DOI:** 10.7759/cureus.107381

**Published:** 2026-04-20

**Authors:** Philip A Giordano, Kurt Weber, Adam Gerber, Andrew Patterson, Steve Carlan

**Affiliations:** 1 Department of Emergency Medicine, Orlando Regional Medical Center, Orlando, USA; 2 Department of Urology, Orlando Regional Medical Center, Orlando, USA; 3 Department of Obstetrics and Gynecology, Orlando Regional Medical Center, Orlando, USA

**Keywords:** agression, emergency cystoscopy, jail inmate, penis surgery, taser

## Abstract

Using conducted electrical weapons (CEW) to incapacitate aggressive, hostile adults as a less-than-lethal approach has become a common law enforcement practice. The most widely used CEW in law enforcement worldwide is the Taser. The target is subdued when an electric circuit is established between two probes, delivering enough energy to cause pain and muscle incapacitation. Injuries and hospitalizations are rare and mainly occur from dart penetration. Removing the dart is usually straightforward and performed by either the target or law enforcement. No previous cases of glans penis injuries from Taser dart penetration have been reported. To our knowledge, no previous cases of glans penis injuries requiring urological surgery from Taser dart penetration have been reported based on a Medline search of the English language literature using the words Taser, case reports, and injury.

A 44-year-old male was struck in the glans penis by an X2 Taser dart during a violent disturbance at the County Jail. He was taken to the Emergency Department because the dart was embedded in his penis just above his distal urethra and was deeply embedded. Urology removed the dart in the operating room by advancing the first barb, cutting it off, then backing out the second barb. The urethra was undamaged. Because of the location of the penetration, the decision-making process for probe removal was challenging.

When taser dart removal is not straightforward, the type of dart and the location of the probe relative to surrounding structures may require changing strategies to safely remove the projectile while protecting the surrounding tissue.

## Introduction

Since 1974, when they were first introduced, conducted electrical weapons (CEWs) have been used by law enforcement as a less lethal method to subdue and control aggressive and violent adults [1]. The most commonly used CEW worldwide is the Taser (Thomas A. Swift’s Electric Rifle, TASER International, Scottsdale, AZ, USA) [2].

The Taser incapacitates targets using two projectiles that deliver pulsed, high-voltage, low-amperage direct current for 45 to 110 microseconds when the circuit is completed between the two probes as they strike the subject [3,4]. 

There are various Taser models with different dart designs, barbs, propellants, ranges, and velocities [5]. They all share a common mechanism of action.

The dart removal usually occurs in the field by trained officers [6]. The dart typically backs out easily with gentle inline traction and rarely requires lidocaine or an incision.

Injuries from Tasers resulting in Emergency Department (ED) visits are rare, and hospitalizations are even less common with estimated rates of ED visits or hospitalizations from 0.25 - 1% [7]. Other data project a prevalence of 4.55 per 1,000,000 EMS [8].

A Taser dart can target any exposed body part, and activation occurs on simultaneous skin contact of both darts. Penetration into sensitive anatomical sites such as the eyes, face, skull, or groin can result in significant complications; therefore, appropriate specialist consultation is recommended when managing injuries involving these areas. Accurate identification of the dart model is essential for planning its removal, and imaging is frequently required for this process. In addition, the decision-making process of removing an embedded dart is complicated by the underlying tissue and the potential for damage from traction on the barb. Taser dart penetration injuries to male genitalia are extremely rare. Previous male penile injury has been reported, but to the best of our knowledge, based on a Medline search of the English language literature using the words Taser, case reports, injury, there have been no documented cases of penile injury requiring urological surgery from a Taser dart to the glans penis. We report a patient with an 11-millimeter double-barbed X2 Taser dart embedded deeply just above his distal urethra. A strategy for removal was difficult because of the angle of penetration directly over the distal urethra and the embedded hook in the glans.

## Case presentation

**Figure 1:**
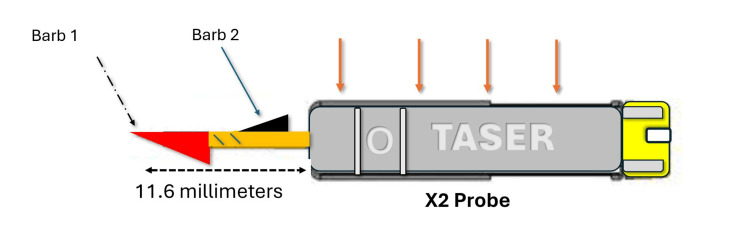
Schematic of the X2 taser probe. The orange lines indicate the groove built into the hub that orients the direction of the barb. Barb 1 (red) is the larger and leading barb (arrow with hatched line). Barb 2 (black) is the following and narrower barb (arrow with solid line). The image was created by the author using PowerPoint (Microsoft, Redmond, WA, USA).

A 44-year-old male was transported to the ED by correctional officers after being subdued and immobilized with a Thomas A. Swift’s Electric Rifle (Taser X2) (Figure 1).

**Figure 2:**
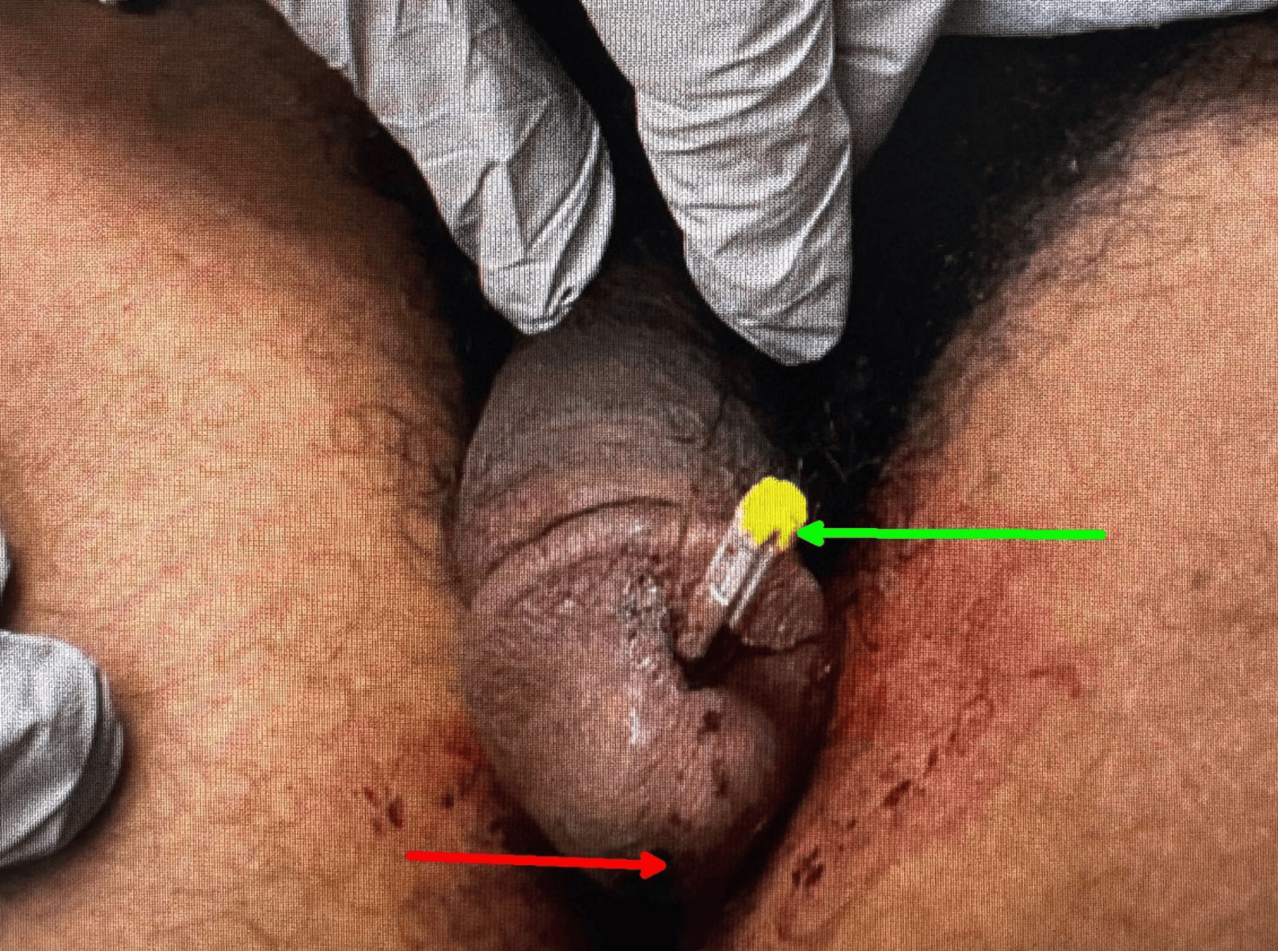
Penis with embedded Taser X2, featuring an 11 mm shaft and barb. Red arrow indicates the external urethral meatus, and green arrow points to the Taser X2 dart.

The 11-millimeter shaft and barb fully penetrated the ventral glans of his penis (Figure 2).

No removal was attempted by the patient or officers. His vital signs were stable, with a blood pressure of 130/86, a pulse of 90 beats per minute, respirations of 19, and a temperature of 98.6 Fahrenheit. His urine drug screen was negative, and he had no microscopic hematuria. His urine drug screen was negative. He had a history of antisocial, aggressive behavior.

**Figure 3:**
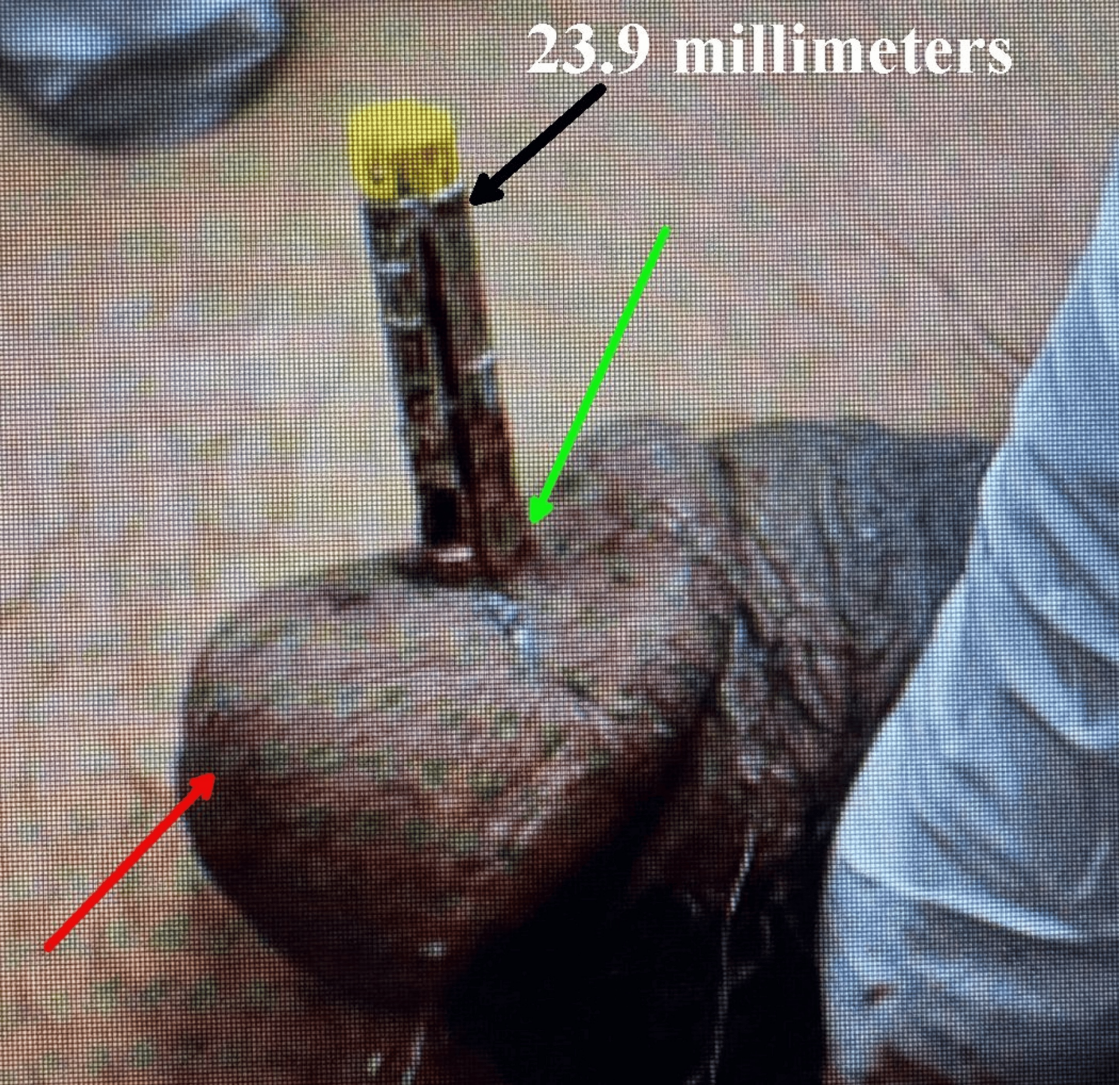
Lateral view of the glans penis showing the X2 dart’s shaft and barb fully embedded. The distance from the bottom of the hub to the tip of the barb concealed in the glans is 11 millimeters. The distance from the top of the hub to the insertion into the glans is 23.9 millimeters (black arrow). The red arrow indicates the urethral meatus, and the green arrow points to the hub-skin interface on the top surface of the glans penis.

During examination, it was determined that the entire barb had penetrated the soft tissue of the glans penis and was embedded directly over the distal urethra. No blood was observed at the urethral meatus, and the patient was able to void only a small amount of urine without gross hematuria. There was no evidence of electric current-related injury or burn. When gentle traction was applied to the probe without anesthesia by the emergency physician, the hub remained flush with the skin, and no part of the shaft was exposed, indicating that the barb tip was lodged at its deepest point. Considering the orientation of the probe's shaft, the soft spongy tissue of the glans, and the uncertainty about the exact depth of the barb tip relative to the urethra, clinical reasoning suggested there was a possibility that the barb was lodged in an area (Figure 3) that could cause urethral damage if moved using standard percutaneous removal methods. 

**Figure 4:**
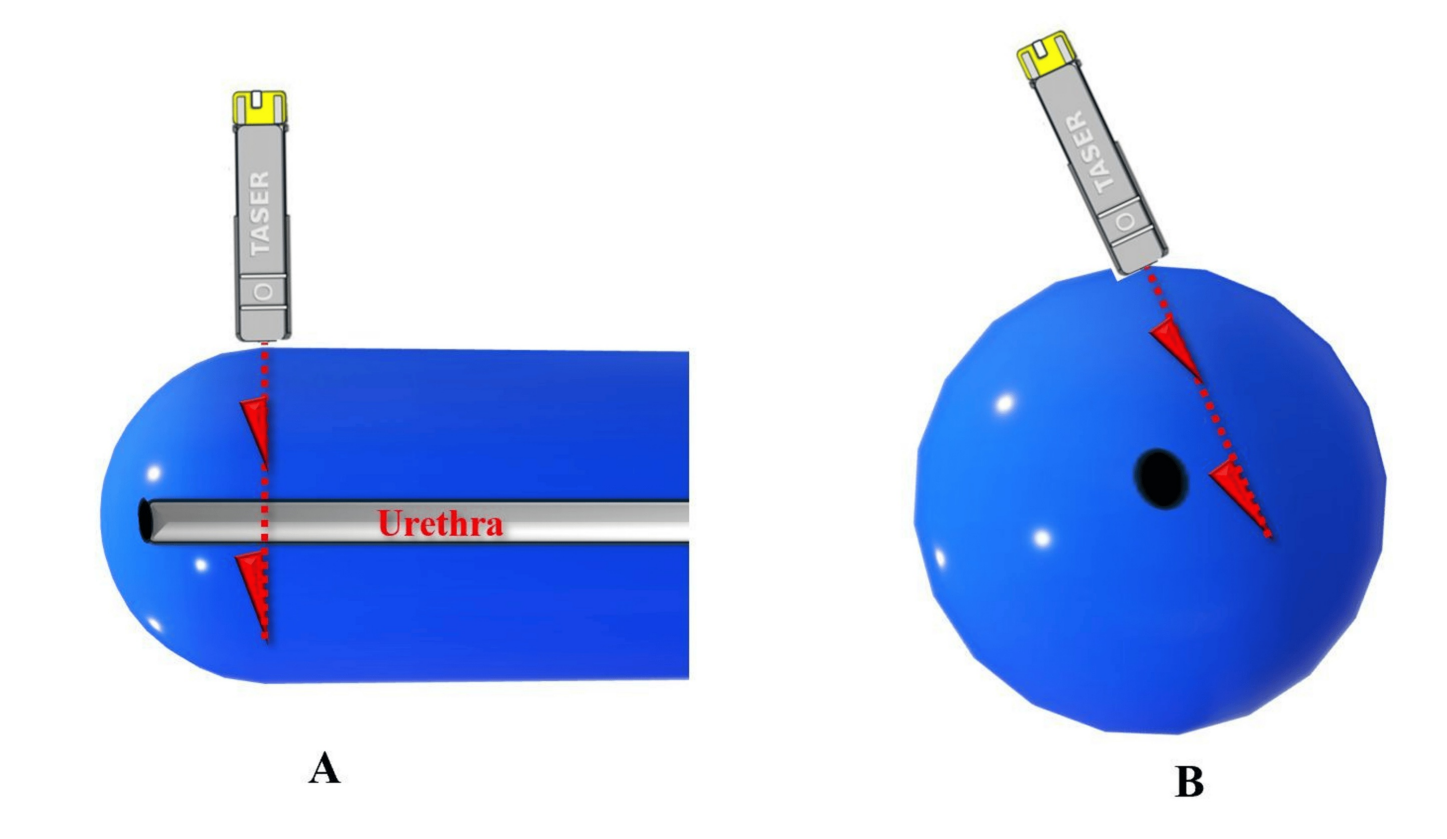
Schematic of the injury. The barb location with respect to the urethra was unknown. ‘A’ and ‘B’ represent longitudinal and cross-sectional representations of the distal penis, respectively. The red dotted line and hooks are the barbs. The black oval is the urethral meatus. No barb could be seen or felt until compression of the glans by the surgeon’s fingers. The image was created by the author using PowerPoint (Microsoft, Redmond, WA, USA).

Therefore, urology was consulted for cystourethroscopy to rule out urethral penetration of the Taser barb before removal. A schematic representation of the barb and urethra can be seen in Figure 4. 

**Figure 5:**
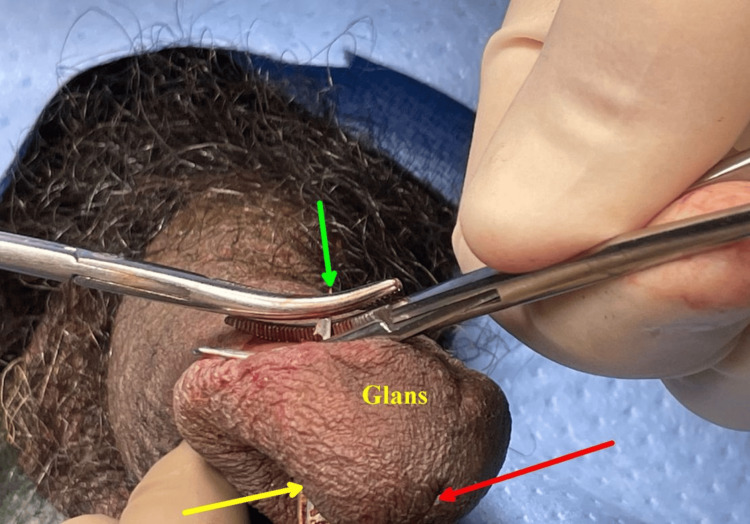
The barb tip is indicated by the green arrow positioned between two hemostats. The yellow arrow points to the hub that is at maximum compression on the glans. The red arrow shows the external urethral meatus.

Urology evaluated the patient in the ED and developed an intervention strategy that included taking him to the operating room for a cystourethroscopy in case urethral repair was needed. After inducing general anesthesia, a short rigid scope was inserted into the urethra. No blood, puncture, or ecchymosis was observed inside the urethra. Since the barb tip was still nearby and pointed toward the urethra, it was decided to redirect the barb away from the urethra and push it out on the ventral sulcus just below the glans and the corona. The hub of the X2 has a metal groove along its side indicating the direction of the barb hook. Once the tip was tenting the skin at this site, a small longitudinal incision was made to expose the barb tip (Figure 5).

**Figure 6:**
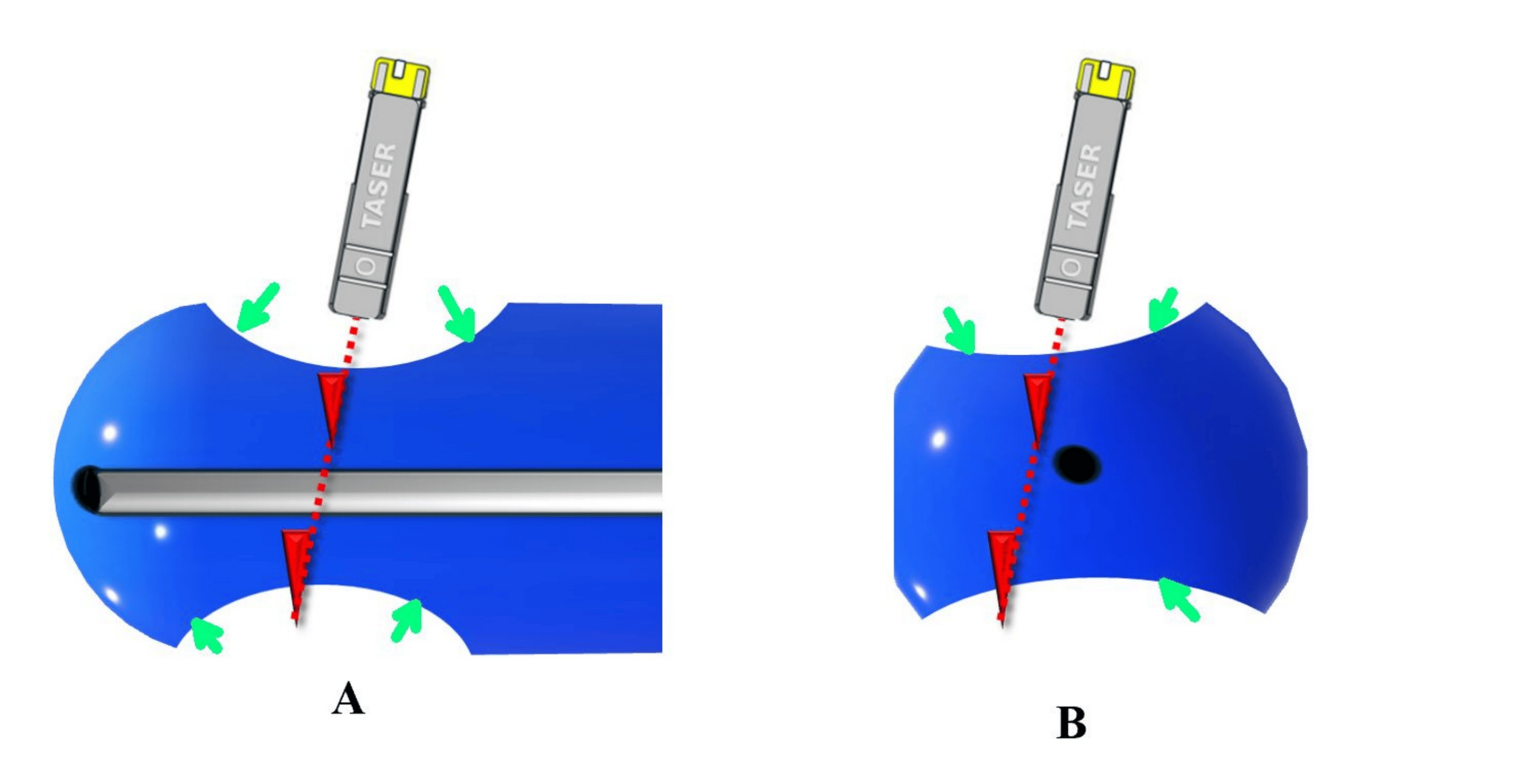
Schematic after compression and before removal of the first barb. ‘A’ and ‘B' represent longitudinal and cross-sectional representations of the distal penis. The red dotted line and hooks are the barbs. The black oval is the urethral meatus. The green arrows are the sites of compression. After compression, the first barb was grasped and cut off. The image was created by the author using PowerPoint (Microsoft, Redmond, WA, USA).

This allowed the skin and underlying soft tissue to be retracted and the larger first barb tip to be exposed (Figure 1, see Barb 1). The barb tip was grasped with a Kelly clamp, then cut with wire cutters. Attempts to further compress the glans between the thumb and index finger to reach the second smallest X2 barb were unsuccessful. Figure 6 is a schematic showing the first barb pushing through the skin with compression and missing the urethra.

**Figure 7:**
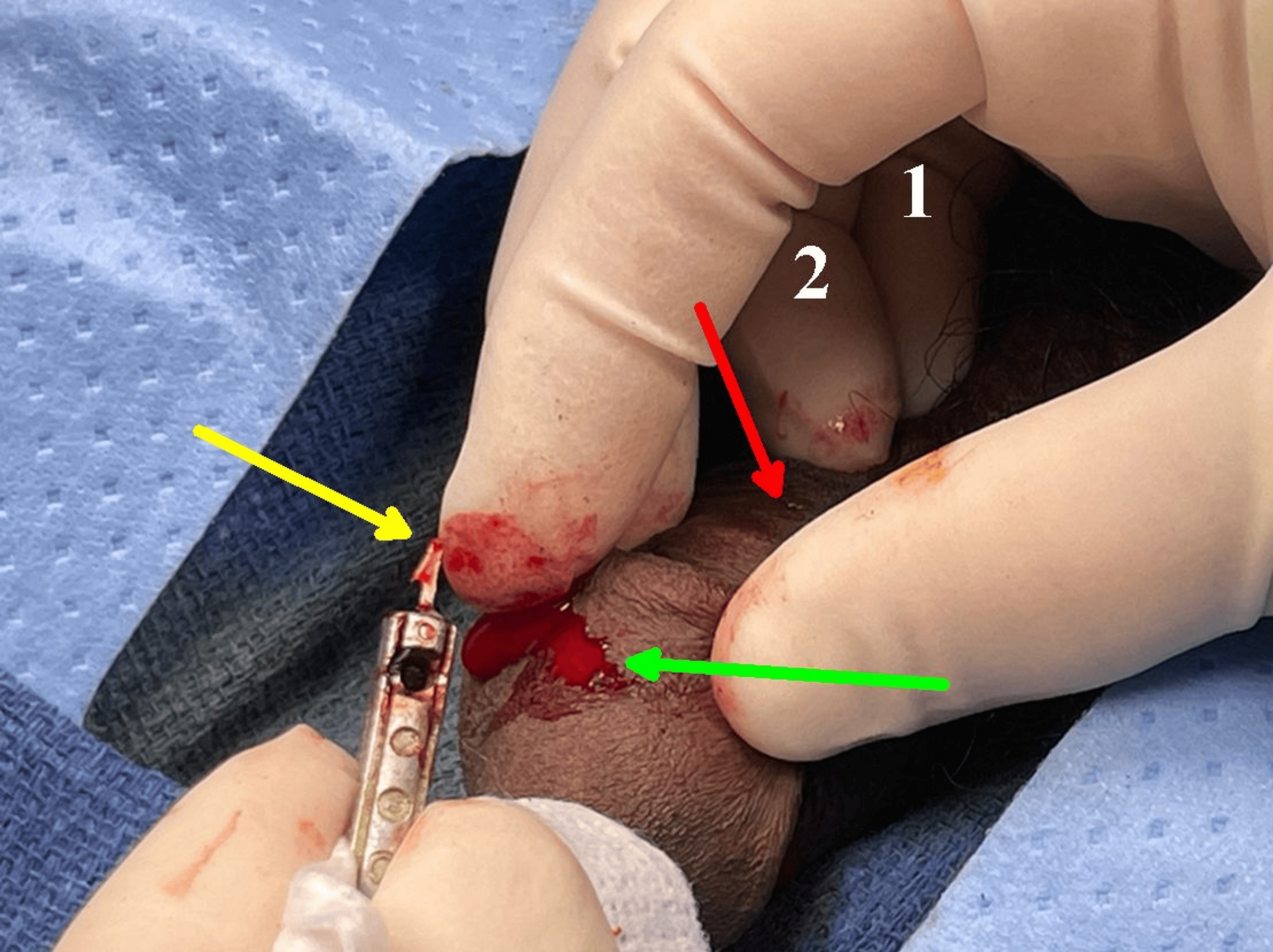
The glans is located beneath the red arrow. The numbers 1 and 2 indicate the surgeon's 5th and 4th digits on the right hand, respectively. The leading, largest barb has been removed. The yellow arrow points to the second barb, and the green arrow marks the barb exit site just below the corona on the shaft of the penis.

Consequently, the rest of the barb was backed out of the dorsal entry site with slight resistance from the smaller proximal barb on the X2 Taser model (Figure 7).

**Figure 8:**
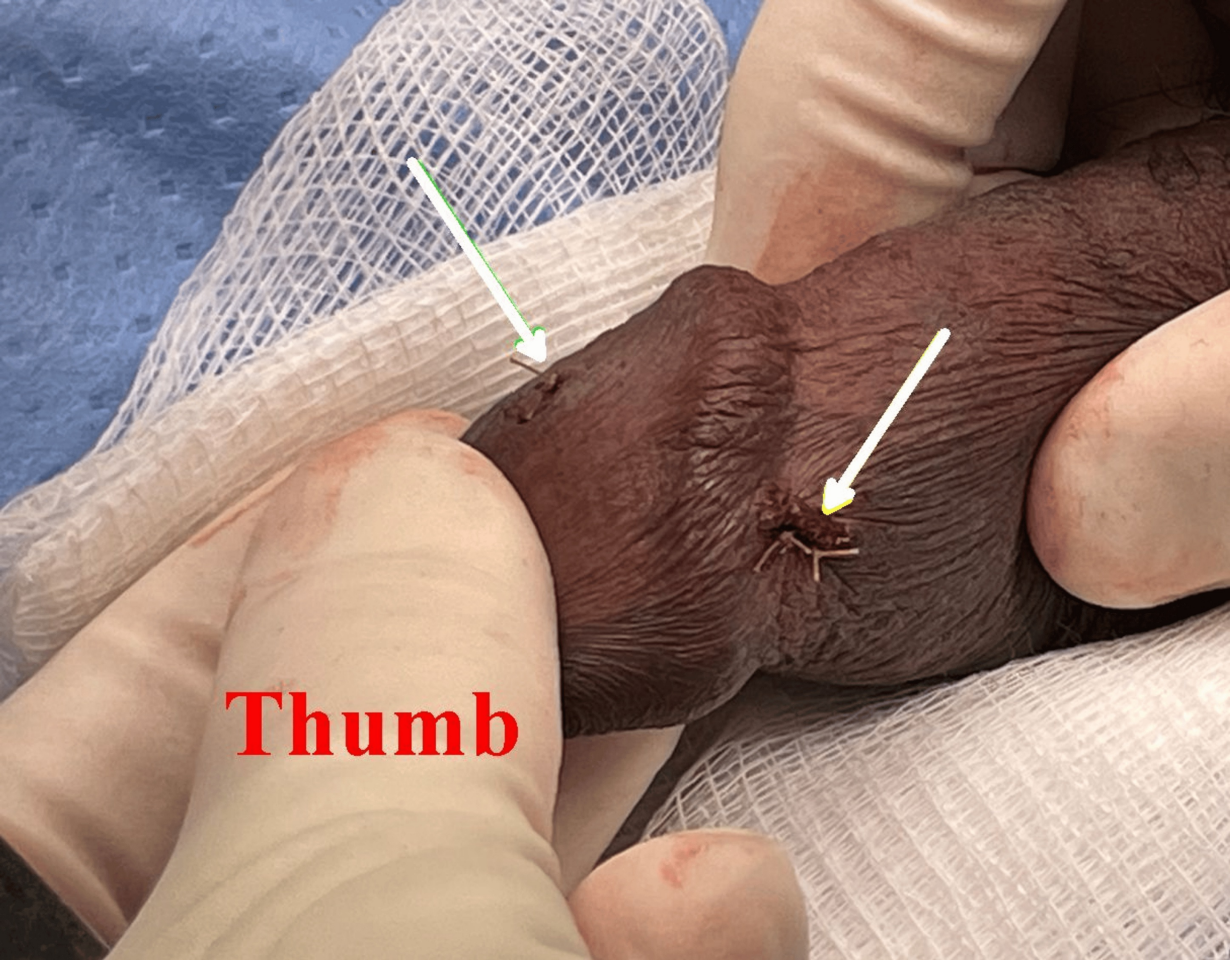
Lateral view of the penis after the procedure. The yellow arrow points to the small skin incision made when the leading probe was pushed through and then cut off. The green arrow indicates both the initial penetration point and the withdrawal point of the second probe.

There was minor bleeding from the ventral sulcus incision, which was controlled with interrupted mattress sutures using 4-0 Chromic Catgut (Figure 8).

**Figure 9:**
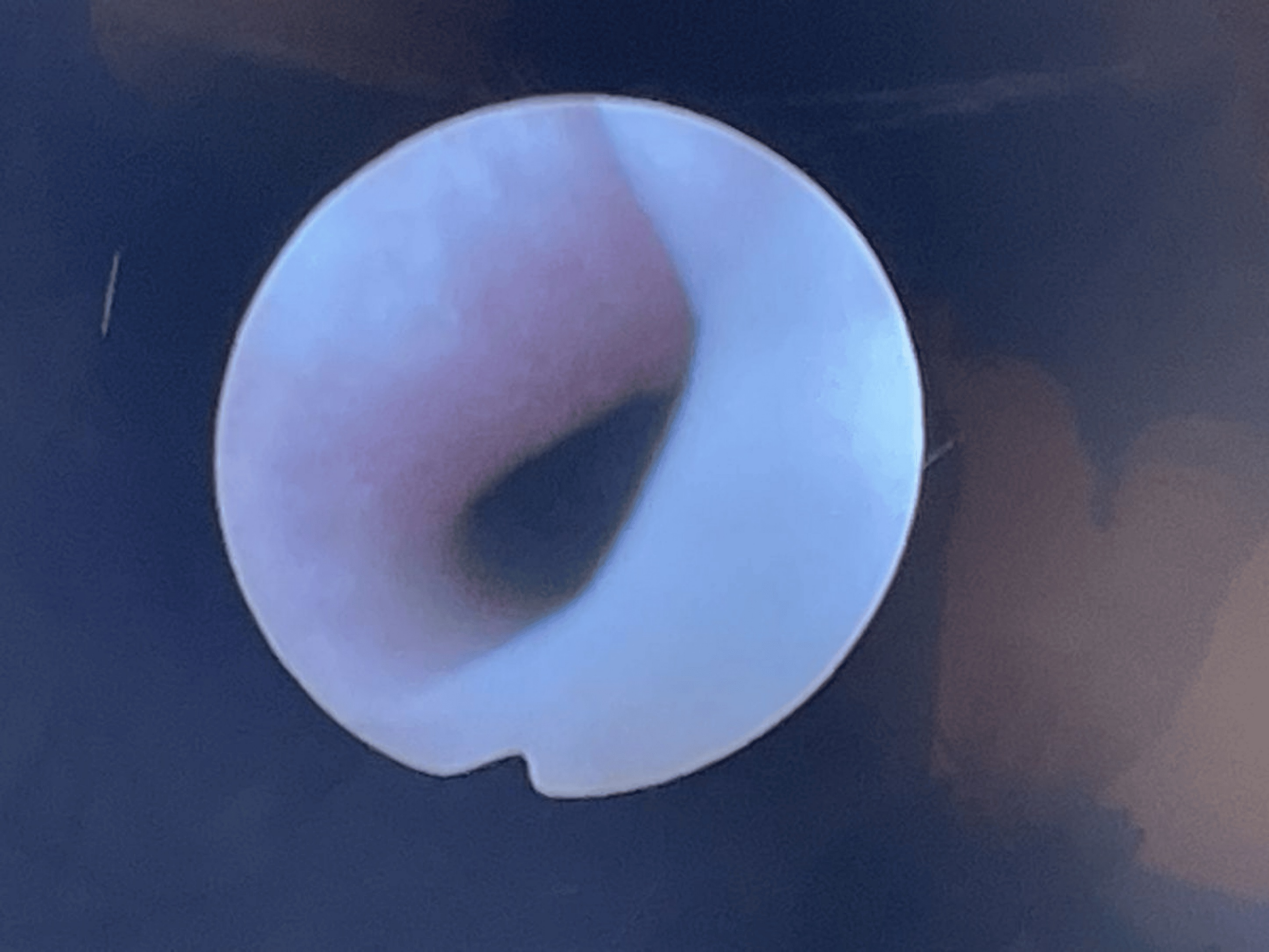
Image of the distal urethra seen on cystourethroscopy at the end of the procedure

The cystourethroscopy was repeated and was normal (Figure 9).

There was no sign of urethral injury. The patient was observed for eight hours after the procedure for signs of hematoma formation. The outcome of his genitourinary health was not obtained, unfortunately, and is a limitation of this report. Patient consent was obtained.

## Discussion

Injuries that occur during efforts to subdue hostile individuals with a Taser are either primary or secondary. Secondary injuries result from involuntary muscle weakness and loss of reflex protective mechanisms, leading to slips, falls, and blunt force trauma [9]. However, serious secondary injuries are very rare [3].

Primary injuries can result from penetration trauma, as observed in our case. Damage caused solely by electric current is generally not known to cause serious health consequences [10]. However, injuries from a Taser dart penetration to various anatomical areas covered with thin skin, which allows deeper dart penetration, have been reported, although they rarely lead to serious complications [1]. Reports include injuries to the skull, face, eyes, fingers, scrotum, throat, chest, and even the shaft of the penis [11]. In 2022, a patient sustained a Taser barb injury to the shaft of the penis during an arrest by police. The shaft had a single barb. The dart was removed in the Emergency Department by cutting along the metal groove that indicates the location of the barb hook. He was observed for two hours post-procedure for evidence of hematoma [11]. Taser dart penetration injury to the scrotum has also been reported. In 2016, a 24-year-old presented to the ED with a Taser dart penetration of his right hemiscrotum. It was manually extracted and scrotal ultrasound revealed a 1.5-cm tract in the mid-right testicle with adjacent hemorrhage. He underwent scrotal exploration, which revealed a puncture through the tunica albuginea with underlying hematoma but no damage to the intratesticular contents [12]. Our case was unique due to the potential for underlying direct urethral damage, which influenced the removal approach. Embedded shafts and barbs are typically removed with gentle traction. Sometimes, lidocaine infiltration is necessary to incise down to the barb with a scalpel for removal. Depending on the barb’s design, it may also be possible to thread a 16-gauge needle over the barb and carefully back it out. For darts embedded in sensitive regions, including the face and groin, an operative or specialist intervention is often recommended [13]. 

Other methods for removing barbs from unusual locations have been described in reported cases [13]. Removal from bone has been described in a case report in 2021. A 30-year-old male had a taser dart embedded in his left clavicle. An ingenious use of a syringe acting as a fulcrum to remove the embedded dart was reported [13].

In our case, cystoscopy was used to verify the absence of urethral injury during removal, particularly due to its position in the glans and the limited tissue available for manual compression. In addition, the conical shape of the glans reduces the amount of intervening tissue between the point of barb entry and the urethra. Only the first large barb was accessible. This case demonstrated that standard ED removal techniques were inappropriate given the two-barb dart design and the orientation of the shaft. Consequently, specialist involvement was clearly indicated. The model of Taser dart, in addition to shaft direction, can influence the removal strategy in embedded darts into the glans. As demonstrated by the embedded bone dart case of 2021, safe removal strategies may require creativity. The key decision point for the ED team in this case was choosing to obtain urological consultation rather than attempting a ‘push-through’ method of dart removal and risk damage to the urethra. Removing embedded darts near sensitive organs risks further tissue injury from barb hooks.

When presented with an embedded taser dart in an otherwise stable patient, a series of steps may be considered. Determine the location of injury recognizing that there are two darts that are discharged into the body. Determine the model number based on inspection of the hub. If the dart is embedded over a sensitive structure obtain imaging or labs to establish the extent of subsurface injury. At this point, the appropriate consultant should be engaged to remove the barb.

In this case, the urethra was undamaged. However, urethral stricture or fistula could result from direct urethral damage.

Recovery typically proceeds without complications. In this case, long-term follow-up is absent and is a major limitation of the paper.

## Conclusions

Direct injury to the glans penis from an embedded X2 Taser barb can potentially involve the urethra. Knowing the model of the dart before attempting removal helps confirm the design of the probe and may assist with extraction. More importantly, the glans penis might not compress enough to allow the base of the second barb to be cut off. Gently backing out the probe with the second barb in place caused no issues. Early urological consultation should be considered when an embedded Taser dart over the glans penis with possible urethral involvement presents for care. Unsuccessful attempts to remove embedded Taser darts over sensitive tissue using standard removal techniques is an indication for specialty consultation.
